# Super-Resolution Reconstruction of Sonograms Using Residual Dense Conditional Generative Adversarial Network

**DOI:** 10.3390/s25216694

**Published:** 2025-11-02

**Authors:** Zengbo Xu, Yiheng Wei

**Affiliations:** School of Textile and Apparel, Shanghai University of Engineering Science, No. 333 Longteng Road, Songjiang District, Shanghai 201600, China; w1hilovemusic1226@outlook.com

**Keywords:** medical ultrasound images, super resolution, generative adversarial network, conditional discriminator network, residual dense module

## Abstract

A method for super-resolution reconstruction of sonograms based on Residual Dense Conditional Generative Adversarial Network (RDC-GAN) is proposed in this paper. It is well known that the resolution of medical ultrasound images is limited, and the single-frame image super-resolution algorithms based on a convolutional neural network are prone to losing texture details, extracting much fewer features, and then blurring the reconstructed images. Therefore, it is very important to reconstruct high-resolution medical images in terms of retaining textured details. A Generative Adversarial Network could learn the mapping relationship between low-resolution and high-resolution images. Based on GAN, a new network is designed, where the generation network is composed of dense residual modules. On the one hand, low-resolution (LR) images are input into the dense residual network, then the multi-level features of images are learned, and then are fused into the global residual features. On the other hand, conditional variables are introduced into a discriminator network to guide the process of super-resolution image reconstruction. The proposed method could realize four times magnification reconstruction of medical ultrasound images. Compared with classical algorithms including Bicubic, SRGAN, and SRCNN, experimental results show that the super-resolution effect of medical ultrasound images based on RDC-GAN could be effectively improved, both in objective numerical evaluation and subjective visual assessment. Moreover, the application of super-resolution reconstructed images to stage the diagnosis of cirrhosis is discussed and the accuracy rates prove the practicality in contrast to the original images.

## 1. Introduction

Medical imaging techniques are commonly used in some disease diagnoses and treatments. It is well known that clear medical images can provide abundant lesion information and improve diagnostic rates. In recent years, ultrasound technology has been developed rapidly in clinical medicine due to its advantages, such as non-invasiveness, non-ionizing radiation, real-time, and convenience. However, low resolution and the high noise of medical ultrasound images (i.e., sonograms) are inevitably caused by imaging systems, imaging environments, and human factors during clinical acquisition. Images are often affected by various factors during the acquisition process (e.g., hardware and software), resulting in degradation and blur, which significantly affects subsequent image processing tasks [1]. Therefore, super-resolution reconstruction technology can be researched to improve the resolution and quality of these medical ultrasound images. Single-image super-resolution is an important task in image processing, which aims to reconstruct a high-resolution image from a low-resolution image and optimize the details and textures to improve the visual perception quality [2].

The classical super-resolution algorithms can be classified into three categories: interpolation-based methods [3,4,5,6,7], reconstruction-based methods [7,8], and learning-based methods [9,10,11,12,13,14,15,16,17,18,19,20,21,22,23,24,25,26].

The interpolation-based methods mainly include linear and nonlinear interpolation, such as Nearest Neighbor (NN), Bilinear, and Bicubic. The prior pixels from the original images are interpolated, and then the interpolated pixels are inserted into the corresponding enlarged images. Although this kind of algorithm is simple and of low complexity, its applicability is not extensive due to unsatisfactory effects. In the reconstruction-based methods, the knowledge of the signal and system is utilized to solve the inverse process of the imaging system, mainly dealing with high-frequency signals. Unfortunately, the cost of computational complexity would be paid. In addition, methods such as image super-resolution, local linear regression, dictionary learning, and sparse coding-based random forest have also been widely used in many fields [3]. Although these traditional super-resolution methods can reconstruct high-resolution images to a certain extent, there are still some problems preventing further research, such as smooth edges and texture details.

In recent years, with the development of machine learning, especially deep learning, it has shown excellent value in super-resolution reconstruction. Deep learning-based SR has significant advantages in learning complex patterns from input data and reconstructing fine texture information, which means improved image quality compared to traditional methods [4]. The pioneering work on super-resolution using a Convolutional Neural Network (SRCNN) can be traced back to [8], where the network consisted of only three convolutional layers. With the simple structure, the multilevel features of the images were not adequately extracted, so that the reconstructed images were blurred. Then Dong [9] improved the SRCNN algorithm and proposed Fast Super-Resolution Convolutional Neural Network (FSRCNN), which deepened the network to 8 layers. The up-sampling method of low-resolution images, i.e., Bicubic, was replaced by deconvolution. The performance of reconstructed images was significantly improved, but the learned features were limited. Ledig [10] proposed SRGAN, which applies GAN for super-resolution reconstruction. In this approach, the Generator (G) learns the features of low-resolution images to generate high-resolution images, while the Discriminator (D) distinguishes the authenticity of the images. On the basis of the weight between G and D, high-quality super-resolution images can be generated. Adversarial loss is considered to achieve better visual performance. Liang et al. proposed SwinIR, which broke the monopoly of CNN by introducing the Transformer architecture. Its feature extraction module is composed of multiple Residual Swin Transformer Blocks (RSTB). And each RSTB combines the Swin Transformer’s localized self-attention mechanism with a residual connection, achieving great performance and efficiency [11]. Chen et al. proposed X-Restormer; this study highlighted the deficiency of task generality in restoration networks, observing that networks perform well on one task and underperform on others. To overcome this problem, they conducted a detailed comparative study across five different restoration tasks to investigate the factors contributing to performance disparity. X-Restormer ultimately demonstrated great task generality and secured state-of-the-art performance in a variety of tasks [12]. Hsu et al. proposed DRCT. DRCT successfully solves the information bottleneck in the Transformer backbone and addresses internal architectural challenges; its core reliance on deep Transformer structures still requires a lot of computational overhead during inference [13]. Chu et al. proposed HMANet. In the field of super-resolution, Swin-Transformer-based models have become mainstream due to their ability to model global spatial information and their moving window attention mechanism, which facilitates information exchange between different windows. This mechanism aims to reduce spatial information loss and stabilize information flow through dense residual connections between layers, thereby unleashing the model’s potential and avoiding information bottlenecks. They achieved outstanding results in the NTIRE-2024 Image Super-Resolution (x4) Challenge [14].

Reconstructing high-resolution medical images provides an effective tool for doctors to find tissue lesions and to detect diseases quickly, as well as improve diagnostic accuracy. Furthermore, technical guidance for the medical images can be provided. At present, most of the reconstruction techniques mainly focus on natural images [7,8,9,10,11,12,13,14,15,16,17,18]. Unlike natural images with bright colors and good visual recognition, medical images are mostly gray images with much more complex textures and rich details, making it difficult to distinguish the regions of interest. Therefore, it is very important to reconstruct high-resolution medical images in terms of retaining textured details. Recently, similar research on medical images has emerged [13,14,15,16,17,18,19,20].

For super-resolution of ultrasound images or sonograms, SRGAN was applied to construct intravascular ultrasound images [19], and could achieve much better performance compared with Bicubic. SRGAN was modified by reducing the number of residual blocks in the Generator for 4 times super-resolution of B-mode images [20]. Lu [21] proposed an Unsupervised Super-Resolution (USS) framework to achieve arbitrary scale factor reconstruction, and experimental results showed that this method was superior to other advanced unsupervised methods. Temiz [22] gave a deep convolutional neural network to realize ultrasonic image super-resolution DECUSR, which was trained with a large number of B-mode ultrasonic image data sets. Wang [23] established a sparse skipping connection U-NET (SSC U-NET) model for ultrasonic image reconstruction, combining two GAN generator models, encoder–decoder model, and a U-NET model. Liu [24] proposed a new super-resolution method for perceptually consistent ultrasonic images based on a self-supervised and cyclic generation adversarial network (CycleGAN), which generated SR images consistent with the original LR images, and the effectiveness of the algorithm was verified with objective and subjective assessments.

Based on the idea of deep learning, this kind of method learns the mapping relationship between low-resolution and high-resolution images. The feature extractors are designed to automatically learn actual ultrasound images, rather than artificial features. Therefore, the reconstructed high-resolution images via this learned knowledge could achieve better quality. Especially, the Discriminator utilized in GAN could distinguish the generated super-resolution images if they are real images, which could further improve the visual quality of these reconstructed images.

But there are also some drawbacks to the sonograms’ super-resolution methods based on GAN. During GAN training, instability is likely to occur, resulting in artifacts in the reconstruction results. The primary purpose of GAN image generation is to facilitate theoretical calculations and provide data support for algorithms, whereas its direct application in clinical diagnosis or training is relatively rare.

Above all, the Residual Dense Conditional Generative Adversarial Network (RDC-GAN) is proposed in this paper to achieve four times super-resolution reconstruction of medical ultrasound images. The main work is as follows:

(1) In the generator, the features of different levels of the original LR image are fully learned and obtained through the cascading of multiple residual dense blocks (RDB), based on the works of SRGAN and Zhang [25]; through global feature fusion (GFF), the hierarchical structure features are adaptively retained in a global way to achieve the use of multi-level information.
(2) In the discriminator, the low-resolution image is used as the condition variable to supervise the generation process of the generator; the feature dimension reduction adopts a 1 × 1 convolution layer instead of the full connection layer, which reduces the calculation amount and increases the nonlinear degree of the network, so as to improve the ability of accurate reconstruction of the network.
(3) A database of 5000 images was established based on some images from the International Symposium on Biomedical Imaging (ISBI) and some images of liver cirrhosis, liver fibrosis, and carotid artery directly provided by the cooperated hospitals. In comparison with some typical reconstruction super-resolution algorithms, our method improves in peak signal-to-noise ratio, structural similarity, and MOS score. In the stage diagnosis of liver cirrhosis, the accuracy and F1 score of mild and severe stages are improved by using reconstructed images.

## 2. Materials and Methods

### 2.1. Designing Scheme

**Figure 1:**
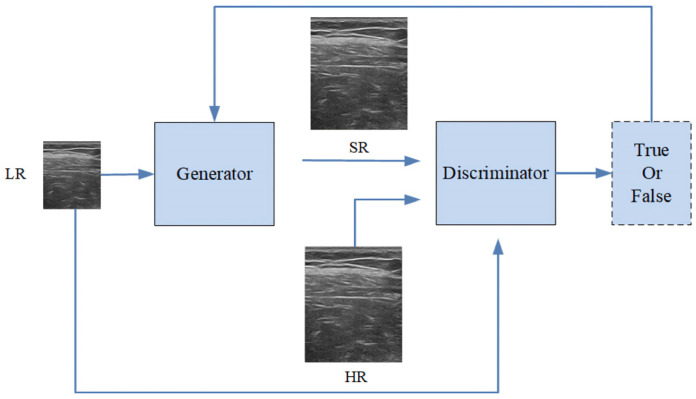
Network flow chart.

Figure 1 presents a general diagram of the proposed method, which consists of the following components: (briefly describe each component here). The remainder of this section provides a detailed explanation of each part of the proposed approach. The whole network consists of the generator network and the discriminator network. The LR images are reconstructed as super-resolution images (SR) by the generator, and then the discriminator determines whether the image is the original high-resolution (HR) image or the generated one (SR). Then the discriminator feedback is delivered to the generator to guide a new generation processing until the whole network becomes stable.

This study proposes several SRGAN-based enhancements to improve feature representation from low-resolution (LR) inputs, thereby improving super-resolution reconstruction performance.

For the generator (G), the Residual Dense network is imported to avoid the simple stacking of convolutional layers. Generally, it is very difficult to extract the features of the LR images directly from each convolutional layer for a deep network. The Residual Dense network could transfer features from convolutional layers through the concatenation of the Residual Dense Blocks (RDB). These hierarchical features are then fused to generate the corresponding reconstructed high-resolution images (SR). Therefore, the hierarchical features from the original LR images are fully learned. Additionally, the Rectified Linear Unit (ReLU) function is employed as the activation function to enhance the network’s performance [27].

For the discriminator (D), the LR image is input as a conditional variable to supervise the generation process. In addition to this, a convolutional layer with a 1 × 1 kernel size instead of a full connection layer is used to control the output information adaptively, which can effectively improve the accuracy of network training.

### 2.2. Generator

**Figure 2:**
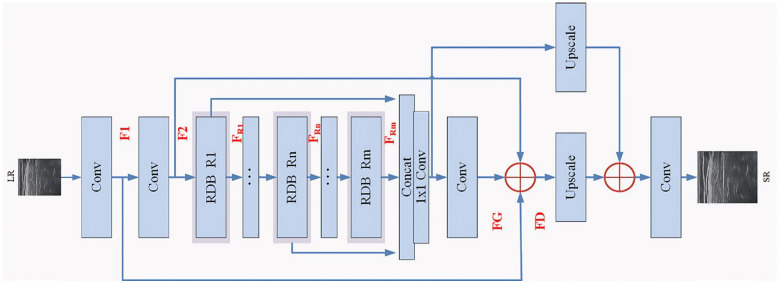
G network architecture.

The architecture of the generator network, illustrated in Figure 2, comprises convolutional layers, residual dense modules, and upsampling blocks. Each residual dense module integrates convolutional operations with ReLU activation [28].

As shown in Figure 2, the LR image is input into the generator network, and then the features from every convolutional layer are extracted by the jumping connections of each layer. The final residual feature *F_D_* before the sampling procedure is obtained as follows:

(1) $$F_{D}=F_{1}+F_{2}+F_{G}$$

where *F*_1_ represents the shallow features extracted from *LR* images by the first convolution operation, and then *F*_1_ is used to extract the feature *F*_2_ through the second convolution operation, as follows:

(2) $$F_{1}=\sigma (W_{1}\times LR+b_{1})$$

(3) $$F_{2}=\sigma (W_{2}\times F_{1}+b_{2})$$

*F*_2_ is used as input to residual dense blocks. Suppose there are *F_G_* is the final global feature fused from all the features of the dense residual module,

(4) $$F_{G}=H_{GFF}\left(\left[F_{R1},\ldots ,F_{Rm}\right]\right)$$

where $H_{GFF}()$ denotes a composite function of 1 × 1 and 3 × 3 convolution, stands for the global feature fusion, is the output of the Rn-th RDB, whose formulas could be checked in Ref. [29].

In short, this is a global residual learning process, and then the hierarchical feature extraction is realized, which tries to make use of multi-level information. To be more specific, there are 20 RDBs and 2 upscale modules in the generator, where, in fact, the upscale module is a deconvolution operation in order to return to the actual image size. Specifically, 64 feature maps are set for all 5 convolutional layers, with 3 × 3 or 1 × 1 kernel size as shown in Figure 2, and the step size is chosen as 1.

### 2.3. Discriminator

**Figure 3:**
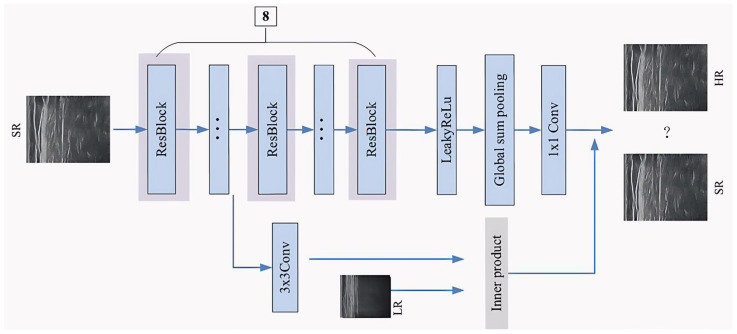
D network architecture.

The discriminator network is shown in Figure 3. It consists of a series of residual blocks [28], the Leaky ReLU activation layers, the pooling layers, and a 1 × 1 convolutional layer, etc. The conditional variable, acting as a regularization on the choice of the discriminator, is introduced into the discriminator by the projection method, which was proposed by Miyato [30,31,32,33]. The generated SR images are input, and then the SR image features are extracted through the first three layers of residual blocks (RB). From the fourth layer, these features are divided into two groups, where the first group is mapped as a 1D vector through a 1 × 1 convolutional layer, and the second group is combined with the LR image as a conditional variable at the fourth layer. The classification labels of the LR image and the convolutional layer of RB are taken to make inner production [33,34]. The values from each group are combined to obtain the final value, which is used directly instead of the probability from the softmax or sigmoid function. Consequently, the final value of D is made by the respective weights of G and D, until it becomes 0.5, and the network remains stable. A convolutional layer with a 1 × 1 kernel size instead of a full connection layer is used to adaptively control the output information. By adjusting the number of 1 × 1 kernels, the channel numbers of feature maps could be increased or reduced flexibly without modifying the feature map size and receptive field, which could introduce much more nonlinearity. Specifically, there are 8 RBs in the discriminator network, and the kernel number is from 64 to 1024 by a factor of 2.

### 2.4. Loss Function

The antagonistic process between G and D in the generative adversarial network can be expressed as follows:

(5) $${min}_{G}{max}_{D}V\left(D,G\right)=E_{HR\sim P_{train}\left(HR\right)}\left[logD_{D}\left(HR\right)\right]+E_{LR\sim P_{G}\left(LR\right)}\left[log\left(1-D_{D}\left(G_{D}\left(LR\right)\right)\right)\right]$$

As for *G*, it is desirable to minimize the *V* value so that the distribution of *G* cannot be recognized by *D*. As for *D*, it is desirable to maximize the *V* value to better identify whether or not the data is classified into the true categories. *E* represents the mathematical expectation of the real data and low-resolution data. The purpose of super-resolution reconstruction is to obtain an image (SR) that is very similar to the original high-resolution image (*HR*).

It is crucial to obtain a much better overall visual performance of the images, not only the evaluation of every single pixel. Therefore, *MSE* and the adversarial loss are selected as the network loss functions in this paper, which are defined as

(6) $$L_{RDC-GAN}=\lambda L_{MSE}+L_{Gen}$$

*MSE* is given by

(7) $$L_{MSE}=\frac{1}{n}\sum_{i=1}^{n}{\left(\frac{1}{W\times H}\sum_{i=1}^{H}\sum_{J=1}^{W}\left(HR\left(i,j\right)-G\left(LR\left(i,j\right)\right)\right)\right)}^{2}$$

where $L_{MSE}$ represents the error loss between *HR* and *G*(*LR*); *G*(*LR*) represents the reconstruction of the *LR* image generation, that is, SR; *W* and *H* represent the width and height of images, respectively; n is the number of training samples.

The adversarial loss is defined as

(8) $$L_{Gen}=-\frac{1}{n}\sum_{i=1}^{n}\left(\frac{1}{W\times H}\sum_{i=1}^{H}\sum_{j=1}^{W}\left(HR\left(i,j\right)\ln D\left(G\left(LR\left(i,j\right)\right)\right)\;\;\;\;\;\;\;\;+\left(1-HR\left(i,j\right)\right)ln\left(1-D\left(G\left(LR\left(i,j\right)\right)\right)\right)\right)\right)$$

where $L_{Gen}$ represents the probability that the reconstructed SR image will be recognized as the true one by the discriminator judgment.

There are two advantages to combining MSE loss with adversarial loss. On the one hand, it can improve the image evaluation of the indicator, peak signal-to-noise ratio (PSNR); on the other hand, it can make up for the high-frequency details that may be lost during the reconstruction process. Specifically, λ is set as 10^2^.

The weighting factor λ was empirically set to 10^2^ to achieve a balance between pixel-level fidelity and texture realism, ensuring both higher PSNR and preservation of fine structural details.

## 3. Results

### 3.1. Experimental Environment

**Figure 4:**
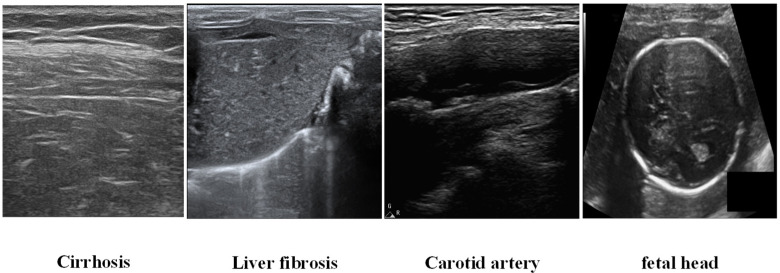
Example images used in the experiments.

There are several data sets used in this experiment, including the fetal head ultrasound images provided by the International Symposium on Biomedical Imaging (ISBI), the images of liver cirrhosis and liver fibrosis of rabbits provided by the ultrasound diagnosis and treatment department of Changzheng Hospital, the Second Military Medical University of the people’s Liberation Army, and the carotid ultrasound images provided by the Central Hospital of Fengxian District, Shanghai. Totally, there are 5000 images in the whole data sets, including 402 liver cirrhosis images, 570 liver fibrosis images, 1527 carotid artery images, and 2501 fetal head images. And 1560 from the set are used for testing, where 30% or 31% images are selected from every subset. The data set, excluding the testing parts, was partitioned with a 9:1 ratio, resulting in 3100 images for training and 340 images for validation. The example images are shown in Figure 4. All experiments are performed with a scale factor of 4× between original low- and high-resolution images.

All the networks are trained on an AMD 3.80 GHz CPU and four GTX1080ti GPUs, totaling 64 G. The TensorFlow deep learning framework is utilized to train the network on the workstation.

### 3.2. Training Process

In this experiment, the mini-batch method is used. Firstly, the 96 × 96 sub-images are obtained by the 8 times random clipping of the original high-resolution images. The low-resolution sub-images with a size of 24 × 24 are obtained by the down-sampled 4× via the Bicubic downsample [28], which is used in the mainstream approaches.

In each training batch, 16 LR images of size 24 × 24 are randomly extracted as inputs. Then, the proposed RDC-GAN with the TensorFlow framework is used and updated with the Adam optimizer. The initial value of the learning rate is set as 1 × 10^−4^ for all layers, and the learning attenuation rate is selected as 0.1. All networks are trained with 3 × 10^5^ update iterations.

### 3.3. Evaluation Criterion

Peak signal-to-noise ratio (*PSNR*), structural similarity (*SSIM*), and MOS scores are used as the evaluation criteria of image quality in this experiment.

*PSNR* is an objective evaluation based on the error between corresponding pixels of two images, where the larger the value, the smaller the distortion. *SSIM* evaluates images in terms of the structure, the brightness, and the contrast. Similarly, the larger the SSIM value, the higher the quality of reconstructed images can be obtained. The corresponding formulas are defined as follows:

(9) $$PSNR=10\times lg\left(\frac{255^{2}}{MSE}\right)$$

(10) $$SSIM=\frac{\left(2m_{HR}m_{SR}+c_{1}\right)\left(2s_{HRSR}+c_{2}\right)}{\left(m_{HR}^{2}+m_{SR}^{2}+c_{1}\right)\left(s_{HR}^{2}+s_{SR}^{2}+c_{2}\right)}$$

where $m_{HR}$ and $s_{HR}$ represent the mean and variance of the original *HR* images, respectively; $m_{SR}$ and $s_{SR}$ represent the mean and variance of the reconstructed *SR* images, respectively; $s_{HRSR}$ represents the covariance between the original images and the reconstructed images; and $c_{1}$ and $c_{2}$ are constants.

MOS score is a kind of subjective evaluation indicator, which describes the overall visual performance by several testers scoring the images.

### 3.4. Experimental Results

In order to verify the performance of the proposed RDC-GAN, comparative experiments are carried out, including the classical super-resolution algorithm Bicubic, and the representative super-resolution algorithm based on deep learning, such as SRGAN and SRCNN. The same data set provided in this paper is used to train all networks and to set the same number of iterations.

**Figure 5:**
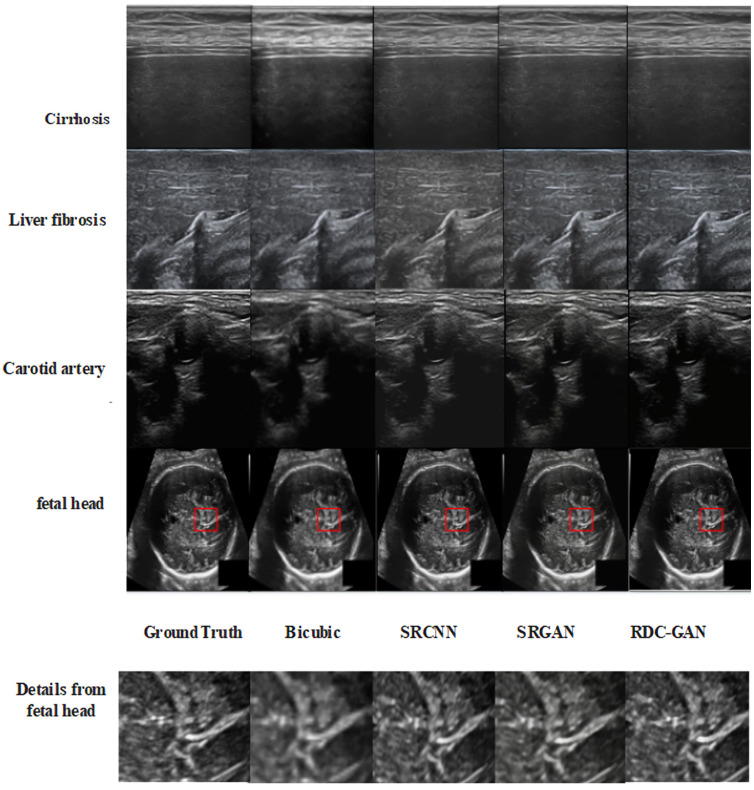
Comparison of super-resolution reconstruction algorithms.

**Figure 6:**
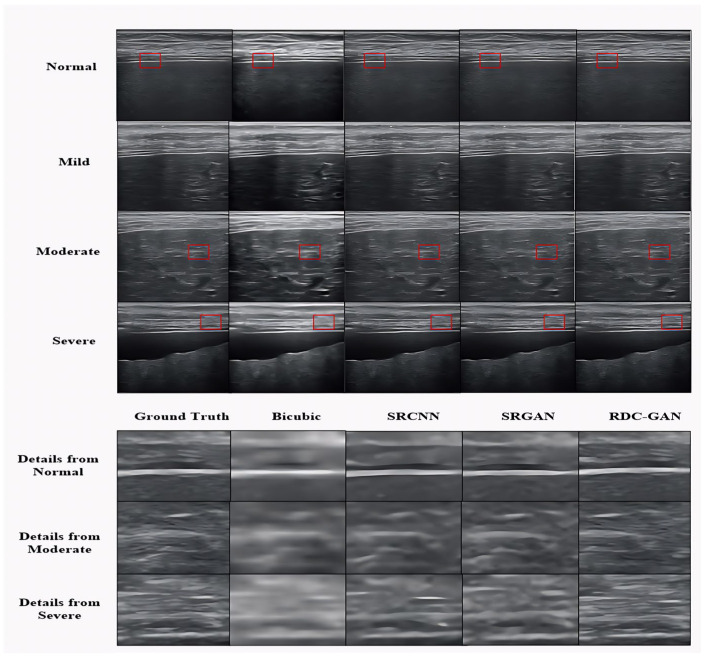
Comparison of algorithms for different stages.

There are four cases in the experiments. The first one is for different kinds of ultrasound images, including cirrhosis, liver fibrosis, carotid artery, and fetal head. The results are shown in Table 1 and Figure 5. The second one is for different cirrhosis stages, including normal, mild, moderate, and severe stages, whose results are shown in Table 2 and Figure 6. The third one is the ablation experiments, and the result is shown in Table 3. The last one is about the computer-assisted diagnostic analysis of cirrhosis for application discussion, and the result is shown in Table 4.

## 4. Discussion

### 4.1. Quantitative Analysis

As can be seen from Table 1 and Table 2, SRCNN, SRGAN, and RDC-GAN are all improved in PSNR compared to Bicubic. Among them, the PSNR and SSIM of SRGANs are lower than those of SRCNN and RDC-GAN. The proposed RDC-GAN algorithm has the highest value of each indicator.

**Table 1:** PSNR and SSIM comparison of various super-resolution reconstruction algorithms.

Image	Algorithm	PSNR (dB)	SSIM (0–1)
Cirrhosis	Bicubic	21.82 ± 0.06	0.75 ± 0.005
	SRCNN	30.29 ± 0.08	0.84 ± 0.003
	SRGAN	25.66 ± 0.07	0.65 ± 0.004
	**RDC-GAN**	**32.55 ± 0.06**	**0.88 ± 0.003**
Liver fibrosis	Bicubic	25.90 ± 0.09	0.80 ± 0.005
	SRCNN	28.94 ± 0.06	0.83 ± 0.003
	SRGAN	26.09 ± 0.07	0.47 ± 0.004
	**RDC-GAN**	**32.87 ± 0.06**	**0.88 ± 0.003**
Carotid artery	Bicubic	24.88 ± 0.07	0.83 ± 0.003
	SRCNN	27.00 ± 0.08	0.79 ± 0.005
	SRGAN	24.57 ± 0.09	0.62 ± 0.003
	**RDC-GAN**	**29.32 ± 0.06**	**0.88 ± 0.003**
Fetal head	Bicubic	25.57 ± 0.05	0.76 ± 0.004
	SRCNN	30.86 ± 0.06	0.86 ± 0.004
	SRGAN	28.00 ± 0.07	0.58 ± 0.003
	**RDC-GAN**	**34.11 ± 0.06**	**0.91 ± 0.003**

**Table 2:** PSNR and SSIM comparison of cirrhosis stages.

Stage	Algorithm	PSNR (dB)	SSIM (0–1)
Normal	Bicubic	20.82 ± 0.07	0.74 ± 0.003
	SRCNN	30.20 ± 0.08	0.85 ± 0.005
	SRGAN	24.97 ± 0.09	0.60 ± 0.004
	**RDC-GAN**	**32.51 ± 0.07**	**0.89 ± 0.002**
Mild	Bicubic	24.31 ± 0.08	0.77 ± 0.003
	SRCNN	31.30 ± 0.06	0.86 ± 0.005
	SRGAN	28.63 ± 0.05	0.78 ± 0.005
	**RDC-GAN**	**32.21 ± 0.08**	**0.87 ± 0.003**
Moderate	Bicubic	21.61 ± 0.09	0.70 ± 0.004
	SRCNN	29.48 ± 0.07	0.78 ± 0.002
	SRGAN	25.10 ± 0.06	0.59 ± 0.003
	**RDC-GAN**	**32.04 ± 0.07**	**0.87 ± 0.003**
Severe	Bicubic	20.52 ± 0.05	0.79 ± 0.004
	SRCNN	30.16 ± 0.06	0.85 ± 0.005
	SRGAN	23.95 ± 0.09	0.63 ± 0.003
	**RDC-GAN**	**33.44 ± 0.06**	**0.87 ± 0.003**

Among them, the average PSNR in the liver cirrhosis data set is improved by 6.78 dB and 3.93 dB, respectively, by comparing with SRGAN and SRCNN, while the other three data sets only increased by 5.92 dB and 2.61 dB on average. It illustrates that in the reconstruction task, it performs better on the liver cirrhosis data set due to its significant structure and feature distinction of each part.

In the super-resolution reconstruction of mild cirrhosis, PSNR (in dB) and SSIM (dimensionless) are used to quantitatively evaluate image reconstruction quality. The PSNR and SSIM reached 32.55 dB and 0.88, respectively. However, the characteristics of liver parenchyma and liver capsule in the image, as well as the texture details of the image, increased due to the increased severity of the disease during the period of moderate liver cirrhosis. Enormous amounts of effective information were lost during the downsampling process. Thus, the effect of the reconstructed image was not improved significantly. In general, the main reason for the improvement of PSNR and SSIM is the addition of residual dense modules and the application of a supervising discriminant model. In the whole process of network learning, the hierarchical features from the LR images are greatly utilized to enrich the details of the image features. The supervision and the guidance of LR images are introduced into D, which restricts the excessive random generation of low-quality images by G, and increases the accuracy of reconstructed images.

### 4.2. Qualitative Analysis

In order to evaluate the overall visual performance of the images, MOS is introduced as a subjective evaluation indicator. The reconstructed images obtained from different super-resolution algorithms are scored by certain testers, and then these images are evaluated by 1~5 scores ranging from 1 to 5. In this experiment, 30 testers were selected for evaluation and analysis, comprising both professional doctors and laypeople. We divided the 30 test subjects into two groups: a group of 10 professional doctors and a group of 20 common people. Each algorithm selects 1500 images (50 × 30 testers) for evaluation and analysis. A score of 1~5 represents image quality, where 1 represents poor quality and 5 represents the best quality. The original images (Ground Truth) are regarded as a standard to set a 5 score.

The evaluation results of both groups are presented in Table 3 below. Based on the data of each group in Table 3, a notable trend was observed in the absolute scores: the professional doctors consistently assigned slightly lower MOS scores than the common group. This phenomenon is consistent with existing real-world image quality assessments, where expert assessors adopt stricter quality standards due to their expertise. However, it is crucial that the overall trend of the four models remains consistent across the two groups.

**Table 3:** Two groups’ MOS.

Algorithm	Professional Doctors	Common People	MOS
Bicubic	2.0	2.2	2.1
SRCNN	3.1	3.3	3.2
SRGAN	3.5	4.1	3.8
**RDC-GAN**	**4.4**	**4.8**	**4.6**

**Figure 7:**
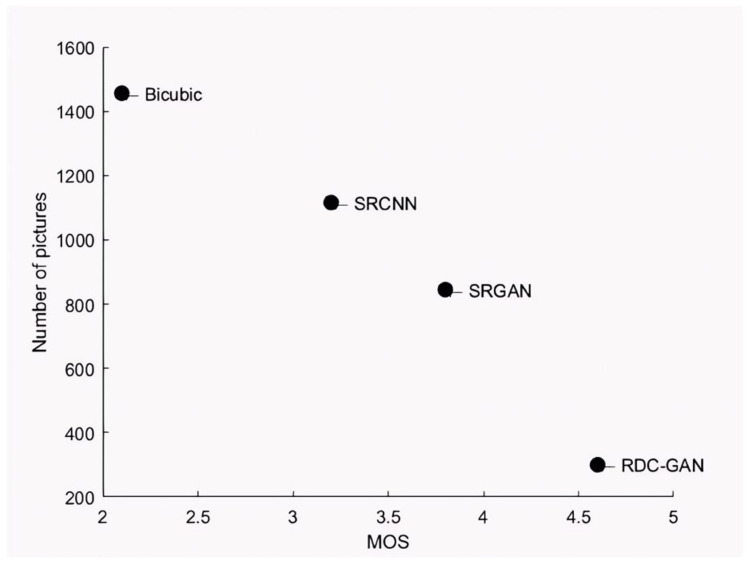
MOS value comparison (50 images × 30 testers, *Y*-axis represents the number of images needed to reach a stable MOS value).

Based on the data of each group in Table 3, we obtain average scores; the average scores of Bicubic, SRCNN, SRGAN, and RDC-GAN are 2.1, 3.2, 3.8, and 4.6, respectively, as shown in Figure 7.

The analysis verifies that the proposed RDC-GAN algorithm is superior to others in overall visual performance. The performance of reconstructed images on cirrhosis and fetal head is shown in Figure 5 and Figure 6, and detailed pictures are the content of the red boxes. The reconstructed images through the traditional Bicubic algorithm are too blurred and have low resolution, based on the visual performance. The algorithm based on deep learning is superior to the interpolation-based algorithms. The performance of SRGAN is better than SRCNN, but some local parts of SRGAN are still blurred. The RDC-GAN algorithm proposed in this paper is similar to the original image, with real details and textures, obvious boundary areas, uniform overall brightness of the image, and the highest MOS score, which achieves a better reconstruction effect. The proposed algorithm does not work well on edge textures in some parts of the data set of the carotid and liver fibrosis. The edge textures are not reconstructed clearly as other algorithms. Thus, the effect of the algorithm effect still needs to be improved.

### 4.3. Ablation Experiments and Analysis

Ablation experiments are conducted, including the RDB (with GFF) and the RDB (without GFF) in the generator network, as well as the projection structure in the conditional discriminant network, to evaluate the experimental performance of the generator structure.

RDB (with GFF) represents that the global feature fusion (GFF) process is contained in the RDB module, while RDB (without GFF) represents that the global feature fusion process is not contained in the RDB module. The six networks are carried out under the same experimental conditions. It can be seen from the table that the networks with no RDB (with GFF), RDB (without GFF), and projection structure have the worst performance (27.93 dB), reflecting that the simple stacked residual layer did not bring good network effect. The network with RDB (using GFF) and projection structure has the best performance, as RDB (with GFF) effectively transfers and fuses the multi-level features of the image. The projection structure allows the conditional variable LR image to guide the discrimination process, ensuring the effective use of information and improving the reconstruction accuracy. The RDB module without the GFF process is applied to the reconstruction task with a slightly improved effect compared with the network with none of the parts; however, it is significantly lower than the RDB module with the GFF process. This is due to the lack of global feature fusion process, where the features are not effectively used.

**Table 4:** Average PSNR for the scale factor ×4 on data set.

	Different Combination of RDB (with GFF), RDB (Without GFF), and Projection					
RDB (with GFF)	×	√	×	×	×	√
RDB (without GFF)	×	×	√	×	√	×
projection	×	×	×	√	√	√
PSNR (dB)	27.93 ± 0.07	31.56 ± 0.08	28.79 ± 0.06	31.47 ± 0.05	31.64 ± 0.06	32.8 ± 0.07

### 4.4. Practical Application Analysis

Based on high-frequency ultrasound images, Liu et al. [3] applied two-level classification idea to stage diagnosis of cirrhosis, where the patch clipping method was used to expand the training set and verify the test set, and the patch block voting principle was adopted in the verification set to obtain the specificity, recall and F1 score of normal, mild, moderate and severe cirrhosis stages. Then, the reconstructed liver cirrhosis data set using the proposed RDC-GAN algorithm (SR, 4 times of OR) was applied to the computer-assisted diagnostic analysis of cirrhosis, with the same experimental conditions.

As shown in Table 5, the application of the super-resolution reconstructed data set (SR) for the classification task increases the specificity by 5.88% and 4.68%, respectively, for mild and moderate cirrhosis compared with the original data set (OR). In terms of recall, the increases are 5.55% and 7.69% for mild and severe cirrhosis. F1 scores are increased by 5.71%, 2.54% and 3.99% for mild, moderate, and severe stages. Some blurred texture details in the original images could be made much clearer by super-resolution reconstruction, and the lesion information becomes more obvious. The accuracy rate has been significantly improved, demonstrating the practicality of the algorithm in real-world applications.

We observed that the inference speed of RDCGAN is highly comparable to that of currently available mainstream super-resolution algorithms, with little difference. RDCGAN strikes an ideal balance between speed and performance, making it potentially suitable for integration into clinical workstations for real-time or near-real-time ultrasound image enhancement.

## 5. Conclusions

To address the issues with medical ultrasound images, such as blurriness, this paper proposes the Residual Dense Conditional Generative Adversarial Network (RDC-GAN). The dense residual modules are added to the network to learn different levels of image features. Furthermore, the LR image is introduced as a conditional variable in the network to achieve better learning features. In order to achieve a better overall image perception effect, the mean square error loss and the adversarial loss are combined. Experiments show that a medical ultrasound image reconstructed four times using the proposed algorithm outperforms Bicubic, SRCNN, and SRGAN in both objective and subjective evaluations, fully illustrating the applicability and validity of the algorithm.

Furthermore, the following work should be investigated in future research: (1) the optimization strategies to generate more accurate super-resolution images; (2) the adaptive magnification instead of the fixed scaling factors to fulfill various scenarios; and (3) the unique super-resolution evaluation indicator for the medical ultrasound images, which could satisfy the overall visual effect and the utility performance with clinical characteristics.

## Figures and Tables

**Table 5:** Sensitivity, specificity, and F1 score in the MA method.

Stage	Specificity/%		Recall/%		F1 score/%	
	OR	SR	OR	SR	OR	SR
Normal	100	100	95	95	97.44	97.44
Mild	94.12	100	88.89	94.44	91.43	97.14
Moderate	84.21	88.89	94.12	94.12	88.89	91.43
Severe	92.31	92.86	92.31	100	92.31	96.3

## Data Availability

The data used in this study involve patient-related medical images and are therefore not publicly available due to privacy concerns and institutional restrictions. Access to the data sets may be granted by the corresponding author upon reasonable request and with permission from the institutional review board.
